# Application of 3D-printed pulmonary segment specimens in experimental teaching of sectional anatomy

**DOI:** 10.1186/s12893-023-02022-6

**Published:** 2023-05-04

**Authors:** Huachun Miao, Jian Ding, Xin Gong, Jian Zhao, Huaibin Li, Kepin Xiong, Xiang Zhou, Wenhui Liu, Feng Wu

**Affiliations:** 1 Department of Human Anatomy, Wannan Medical College, Wuhu, China; 2 Department of Cardio-Thoracic Surgery, The First Affiliate Hospital of Wannan Medical College, Wuhu, China; 3 Shandong Digital Human Technology Co., Inc., Jinan, China

**Keywords:** Pulmonary segment, 3D printing, Sectional anatomy, Education

## Abstract

**Background:**

Lung cross-section is one of the emphases and challenges in sectional anatomy. Identification of the complex arrangement of intrapulmonary tubes such as bronchi, arteries, and veins in the lungs requires the spatial imagination of students. Three-dimensional (3D) printing has become increasingly used in anatomy education. This study aimed to analyze the effectiveness of 3D-printed specimens used for the experimental teaching of sectional anatomy.

**Methods:**

A digital thoracic dataset was obtained and input into a 3D printer to print multicolor specimens of the pulmonary segment after software processing. As research subjects, 119 undergraduate students majoring in medical imaging from classes 5–8 in the second-year were chosen. In the lung cross-section experiment course, 59 students utilized 3D printed specimens in conjunction with traditional instruction as the study group, while 60 students received traditional teaching as the control group. Preclass and postclass tests, course grading, and questionnaire surveys were used to assess instructional efficacy.

**Results:**

We obtained a set of pulmonary segment specimens for teaching. The students in the study group scored better in the postclass test than those in the control group (*P* < 0.05), and the students in the study group scored higher in satisfaction with the teaching content and spatial thinking for sectional anatomy than those in the control group (*P* < 0.05). The course grades and excellence rates in the study group exceeded those in the control group (*P* < 0.05).

**Conclusion:**

The application of high-precision multicolor 3D-printed specimens of lung segments in experimental teaching of sectional anatomy can improve teaching effectiveness and is worth adopting and promoting in sectional anatomy courses.

## Background

Medical imaging is an indispensable tool in disease research and diagnosis, and sectional anatomy is the morphological basis for the observation and analysis of tomographic images [1, 2]. Experimental teaching is the cornerstone of sectional anatomy. It is necessary to establish the tomographic thinking of “from whole to cross-section and from cross-section to the whole”. Students must be able to follow and observe consecutive tomographic specimens based on in the mastery of stereological structures [3], this could be the only way in which medical images can be interpreted correctly. The sectional anatomy of the lung is one of the emphases and challenges in the course; the complex arrangement of intrapulmonary structures such as bronchi, arteries, and veins in the lungs requires high spatial imagination in students. The identification and understanding of the anatomical tomography of the pulmonary hilum and segments are necessary to learn how to diagnose pulmonary diseases with medical imaging. The time for teaching a specific course is limited, and finding a way to increase teaching effectiveness within a certain amount of time is the main goal that instructors in medical colleges and universities always pursue.

Three-dimensional (3D) printed models are an excellent teaching resource in anatomy education [4], which are useful tools for studying normal, uncommon, and pathological anatomy [5]. Such models can facilitate the visuospatial comprehension of sectional anatomy [6, 7]. Currently, 3D printed models of bronchial trees have been reported to facilitate novice learning of radiology anatomy [8], but models of lung segments integrating bronchial trees, pulmonary arteries, and veins have not been reported to be applied in teaching. In this study, 3D printed specimens of lung segments were applied to the experimental teaching of pulmonary sectional anatomy, and the teaching effects were evaluated.

## Methods

### Research subjects

The subjects of the study were undergraduate students of medical imaging in second-year classes 5, 6, 7, and 8 at Wannan Medical College. Fifty-nine students in classes 7–8 composed the study group, and 60 students in classes 5–6 composed the control group. There were 21 male and 39 female students in the control group, with a mean age of 20.27 ± 0.87 years, and 19 male and 40 female students in the study group, with a mean age of 20.18 ± 0.92 years. There were no statistically significant differences in gender(P = 0.847) and age(P = 0.624) between the two groups.

### Printing of the pulmonary segment specimens

**Fig. 1:**
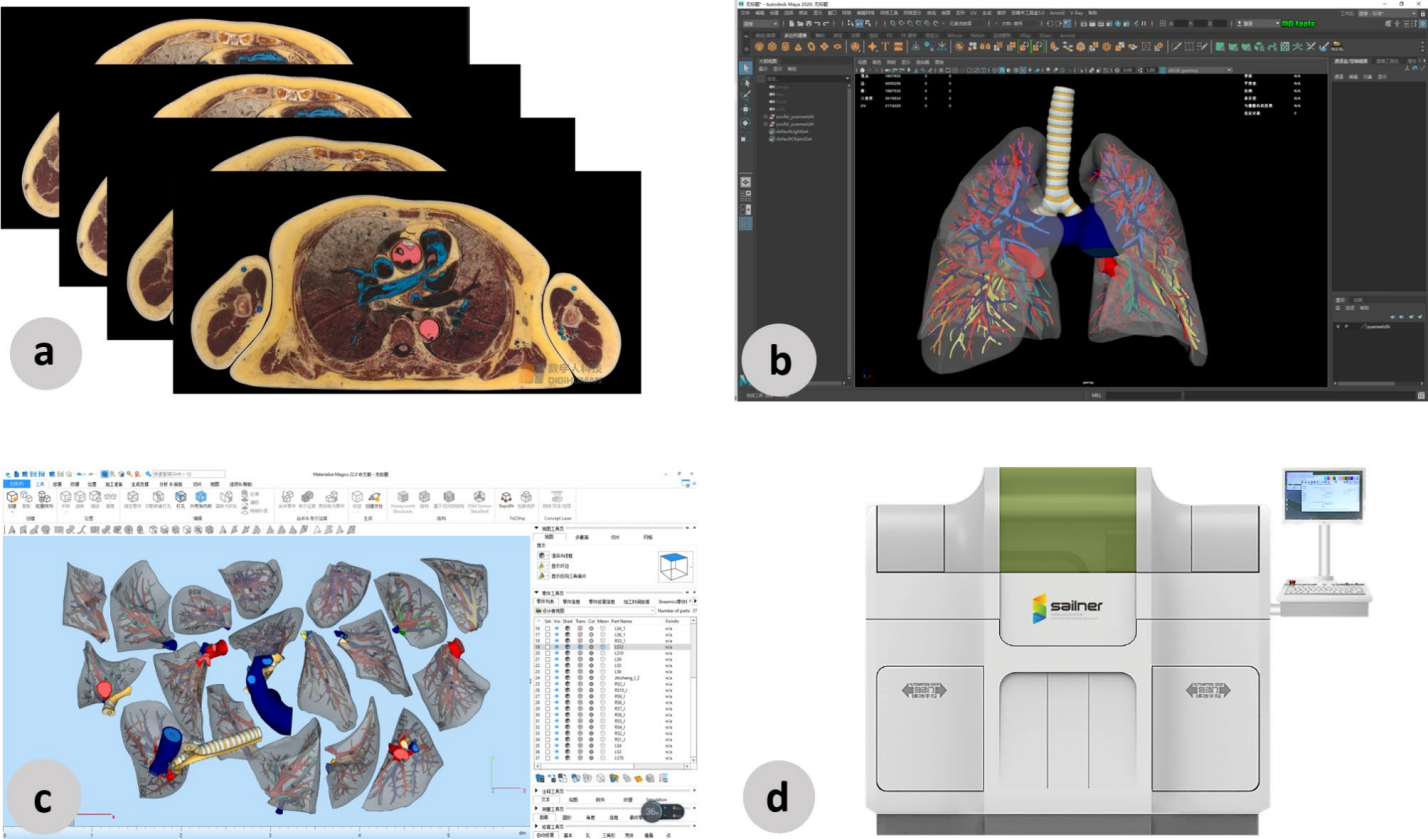
The printing process of the pulmonary segment specimens. **a** The digital thoracic dataset. **b** The 3D digital model after segmentation and modification displayed via Maya software. **c** The isolated bronchopulmonary segments displayed via Magics software. **d** The full-color 3D printer

A female digital thoracic dataset from Shandong Digital Human Technology Co., Inc., with a voxel size of 0.0384 mm*0.0384 mm*0.1 mm was chosen [9]. The segmentation data were obtained by manually delineating the boundaries and tissue structures in Photoshop software. An improved Marching Cubes algorithm was then used to reconstruct the 3D digital models [10]. The segmentation data were repeatedly modified and validated, ensuring their accuracy. The data obtained were optimized in the software Maya and Magics using tools such as rewiring, thickness adjustment, and hole repair. Finally, the model was split according to the segment of the bronchus (Fig. 1a, b and c). All segmentation and postprocessing were performed by an engineer from Shandong Digital Human Co., Inc., China. The digital models were verified for anatomic accuracy by anatomic experts and a senior cardiothoracic surgeon.

The data were imported into a 3D printer (J401Pro, Zhuhai Sailner Technology Co., Ltd., China) to create the specimens. The printer can print full-color models in seven different materials for medical use (Fig. 1d). After printing, the split parts were made into magnet slots, and the magnets were placed for assembly.

### 3D printed model

**Fig. 2:**
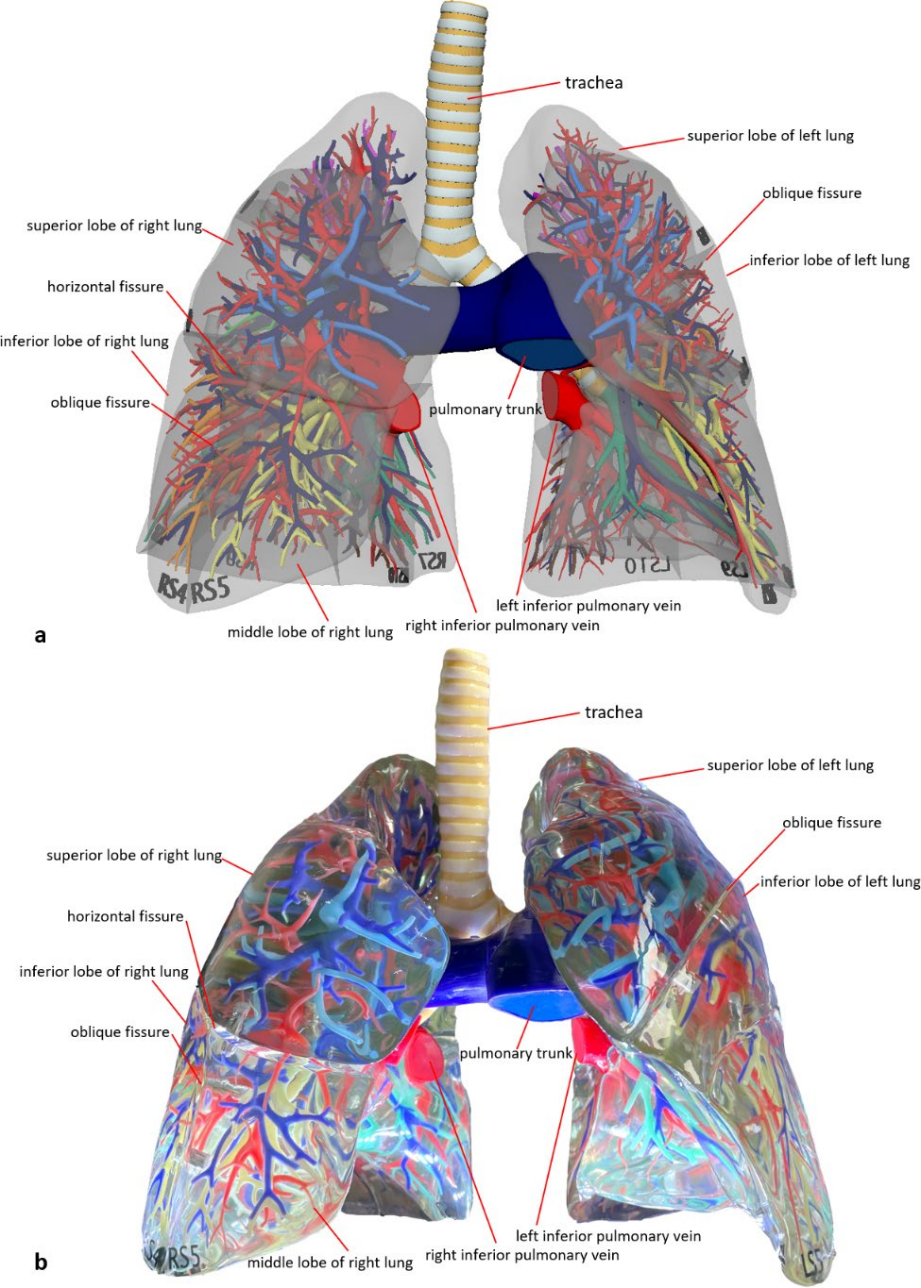
A holistic view of the 3D-printed lung specimens. **a** 3D digital model of the lungs. **b** 3D-printed specimen of the lungs

**Fig. 3:**
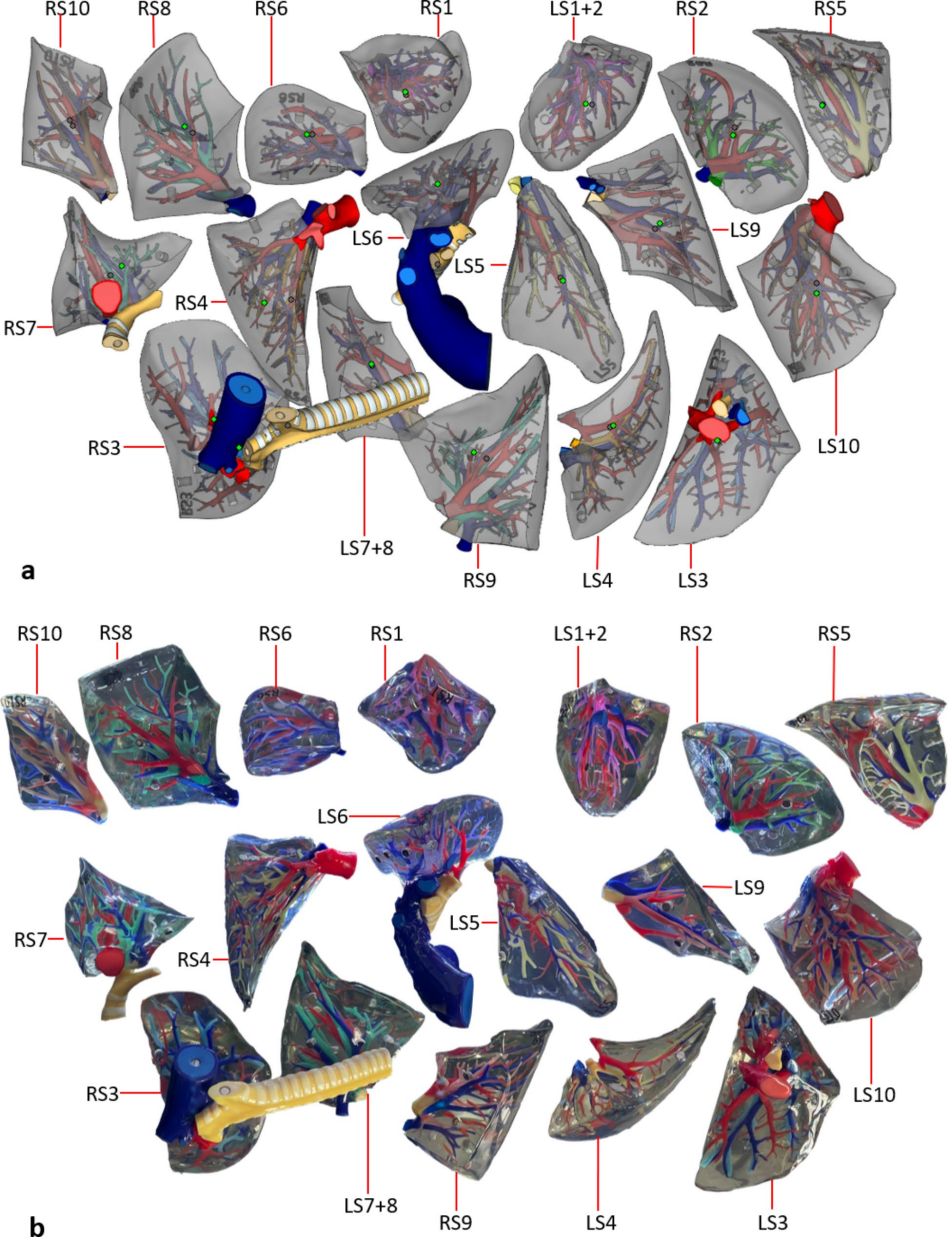
Pulmonary segment specimens. **a** 3D digital model of the pulmonary segments. **b** 3D printed specimen of the pulmonary segments

We obtained a set of lung specimens printed in full color at a 1:1 scale based on high-definition digital human anatomy data. It was the same size and shape as real lungs. The lung parenchyma was printed with transparent material, and the bronchial tree was colored; the bronchi on each side of the lung segment were distinguishable by distinct colors, while the pulmonary segments with the same name on both sides had the same color, for a total of 18 submodels. Magnets were absorbed into each submodel to provide a detachable and integrated display of lung parts (Figs. 2b and 3b).

### Teaching methods

This session of the sectional anatomy experimental course concerned lung cross-section. Two groups of students were successively taught in the same laboratory, under the supervision of two identical professional instructors. The syllabus outlined identical instruction time and content for both groups. Initially, multimedia presentations were used to teach the lung segments and intrapulmonary ducts (Table 1).

In the control group, with the guidance of the sectional anatomy atlas and textbook, slicer knives were used to transect isolated lungs. This produced lung slices in accordance with the ten standard cross-sections of the lung found in the textbook. The lung segments and their key structures were then observed and studied by comparing them with computed tomography (CT) and magnetic resonance imaging (MRI) images.

**Fig. 4:**
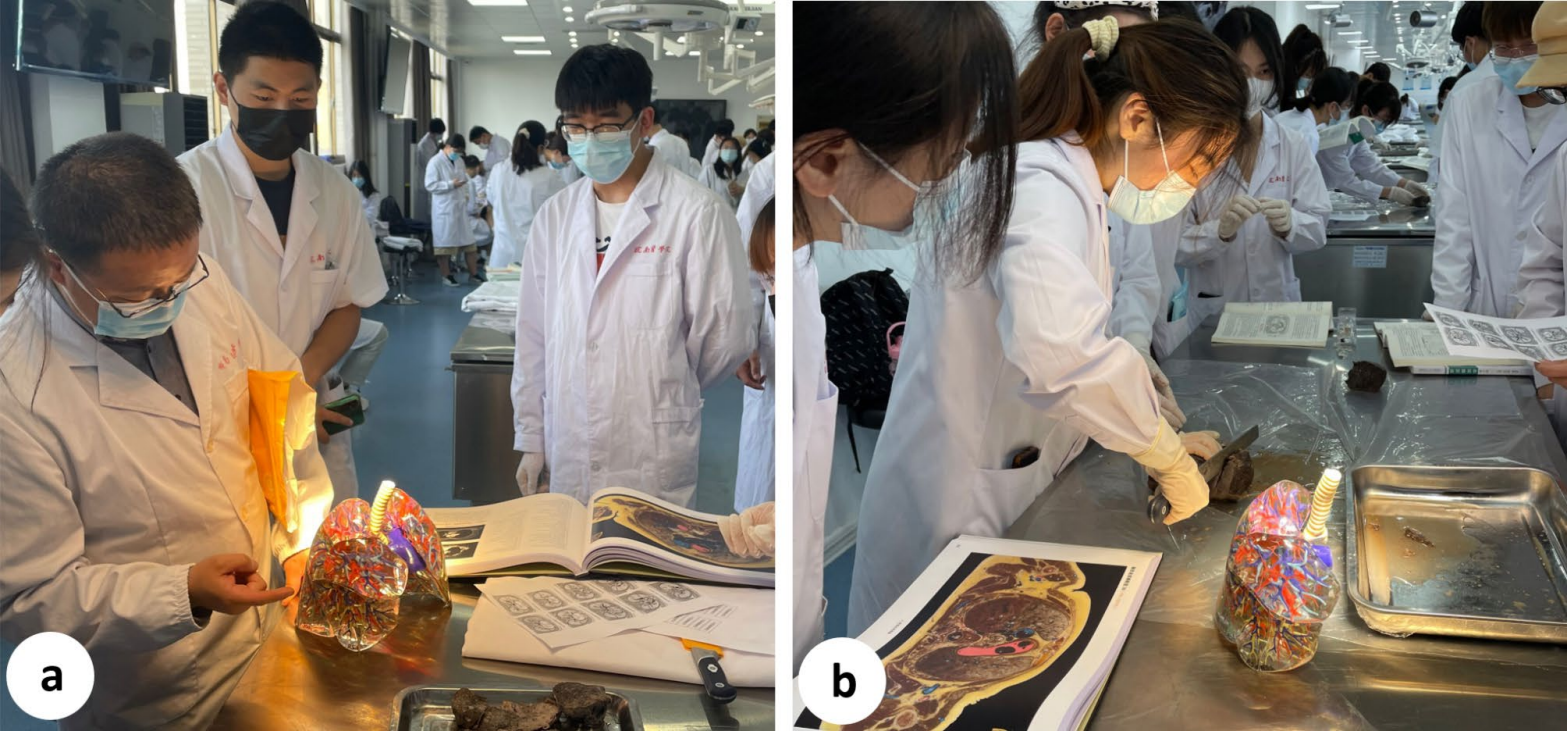
Application in sectional anatomy experimental teaching. **a** The teacher explained the specimen in the laboratory. **b** Students performed experiments with the guidance of the specimen

Students in the study group were additionally taught using 3D printed specimens of lung segments. The overall structure of the lung was observed, and the position and shape of each lung segment were examined from different angles, including the rib surface, mediastinal surface, and diaphragm surface (Fig. 2). These lung specimens were divided into eighteen segments, and the morphology of each lung segment, including the bronchi, arteries, and veins, was studied (Fig. 3). With guidance from the 3D printed specimens and the sectional anatomy atlas, students used the slicer knives to create transverse sections of the lungs to identify and understand key structures in the sections (Fig. 4). They also compared their observations with medical images.

Overall, both groups of students received the same instruction time and content, but the study group used 3D printed specimens in addition to the traditional methods used by the control group.

**Table 1:** Bronchopulmonary segments of the right and left lungs

Lung	Lobe	Segment	Serial number
Right lung	Superior lobe	Apical segment	RS1
		Posterior segment	RS2
		Anterior segment	RS3
	Middle lobe	Lateral segment	RS4
		Medial segment	RS5
	Inferior lobe	Superior segment	RS6
		Medial basal segment	RS7
		Anterior basal segment	RS8
		Lateral basal segment	RS9
		Posterior basal segment	RS10
Left lung	Superior lobe	Apicoposterior segment	LS1 + 2
		Anterior segment	LS3
		Superior lingular segment	LS4
		Inferior lingular segment	LS5
	Inferior lobe	Superior segment	LS6
		Medioanterior basal segment	LS7 + 8
		Lateral basal segment	LS9
		Posterior basal segment	LS10

### Teaching effect evaluation

Both groups of students were given a preclass test before the experimental session and a postclass test and questionnaire survey after the session. The preclass and postclass assessments had the same number of questions and knowledge point difficulty. There were ten fill-in-the-blank questions, including the lung segment location; lung segment bronchus, arteries and veins; lung segment on cross-section; and identification of key structures in CT images. The contents of the subjective questionnaire survey were designed and are shown in Table 2, including an understanding of the morphology and location, the pipeline, the cross-sections, the CT tomography of lung segments, the spatial thinking of sectional anatomy, and the overall satisfaction with the teaching. After the course, the course grade, including the final exam scores, was collected on a percentage scale and used to assess the effectiveness of the instruction.

**Table 2:** Evaluation of student satisfaction with the teaching effectiveness using the Likert Scale Questionnaire. 5-point Likert scale: ①Strongly dissatisfied, ②Dissatisfied, ③Neutral, ④Satisfied, ⑤Very satisfied

Question	Strongly dissatisfied	Dissatisfied	Neutral	Satisfied	Very satisfied
1. Understanding of lung segment location and morphology	①	②	③	④	⑤
2. Understanding of the pipeline within the lung segment	①	②	③	④	⑤
3. Understanding of the lung segments on the cross-sections	①	②	③	④	⑤
4. Understanding of the CT tomography of lung segments	①	②	③	④	⑤
5. Development of spatial thinking for sectional anatomy	①	②	③	④	⑤
6. Overall satisfaction with teaching	①	②	③	④	⑤

### Statistical processing

SPSS 18.0 software was used for data analysis. A t-test was used for comparisons between the two groups. Counting data were expressed by examples or percentages, and the chi-squared (χ2) test was used for intergroup comparisons.

## Results

### Results of pre- and postclass tests

The results revealed that there was no statistically significant difference in the precourse test scores of the two groups (*P* = 0.261). The postclass test scores in both groups were higher than the preclass test scores, and the incremental value in the study group was significantly greater than the increase in the control group (all *P* < 0.001; Table 3).

**Table 3:** Comparison of pretest and posttest scores between the two groups

	Study group (*N* = 59)	Control group (*N* = 60)	T value	*P* value
Preclass test (points)	5.88 ± 1.74	5.51 ± 1.76	1.129	0.261
Postclass test (points)	8.36 ± 1.36	6.97 ± 1.54		
T value	10.480	8.346		
*P* value	<0.001	<0.001		
Incremental value (points)	2.48 ± 1.81	1.45 ± 1.35	3.504	<0.001

### Evaluation of the teaching effect

All students in this study completed the subjective questionnaire survey. As shown in Table 4, the comparative analysis between both groups showed that there was no difference between both groups in terms of understanding the morphology and location of the lung segments (*P* = 0.099). However, regarding understanding the ducts within lung segments, identification of lung segments on cross-section and CT tomography, developing spatial thinking for sectional anatomy, and overall satisfaction with the course, the study group was significantly more satisfied than the control group (all *P <* 0.001; Table 4).

**Table 4:** Comparison of the teaching satisfaction between the two groups using the Likert Scale Questionnaire

Question	Study group (*N* = 59)	Control group (*N* = 60)	T value	*P* value
1(points)	3.98 ± 1.20	3.67 ± 0.83	1.661	0.099
2(points)	4.03 ± 1.19	3.28 ± 1.20	3.395	<0.001
3(points)	3.95 ± 1.14	2.97 ± 1.30	4.335	<0.001
4(points)	4.03 ± 1.01	2.70 ± 0.84	7.772	<0.001
5(points)	4.10 ± 0.97	2.75 ± 0.98	7.514	<0.001
6(points)	4.02 ± 0.96	3.07 ± 0.69	6.153	<0.001

### The final rating of the course

The results suggest that the scores and the excellence rate of the students in the study group were significantly higher than those of the control group, and the differences were statistically significant (both *P* < 0.05; Table 5).

**Table 5:** Comparison of the course scores and grade scales between the two groups

	Study group (*N* = 59)	Control group (*N* = 60)	T(χ2) value	*P* value
Course grade (points)	87.88 ± 12.73	83.43 ± 13.40	2.051	0.042
Excellent (n, %)	39(66.10%)	26(45.00%)	11.77	0.019
Good (n, %)	6(10.17%)	17(26.67%)		
Medium (n, %)	8(13.56%)	4(6.67%)		
Pass (n, %)	5(8.47%)	11(18.33%)		
Fail (n, %)	1(1.69%)	2(3.33%)		

## Discussion

3D reconstruction based on cross-sectional anatomy began in the middle 1990s with the Visual Human Project (VHP) as a resource for teaching human anatomy [11]. The Virtual Human Dissector software, which was developed from the VHP, can help students interpret cross-sectional images and understand the relationships between anatomical structures [12]. Browsing software based on Visible Korean data was used to teach sectional anatomy and was a valuable tool to teach medical students [13]. Based on China’s Digitalized Visible Human (CDVH) data, an anatomy assistive teaching system was developed that plays a positive role in guaranteeing the effect and quality of anatomy teaching [9]. The digital thoracic dataset in our study was obtained from the CDVH high-resolution datasets, and the reconstructed digital model was rich in detail.

There are three well-accepted methods of 3D representations: 3D printing, virtual reality (VR) glasses, and 3D display [14]. VR glasses and 3D displays require terminal equipment and do not mesh well during experimental operation. 3D-printed specimens are convenient for demonstrating anatomical intricacies and spatial order during the operation stage. 3D-printed lung and bronchial tree models could be applied as simulators for surgical training and preoperative planning [15–17]. In recent years, the application of high-precision 3D models in medical teaching has enabled students to visually observe the fine structure and position relationship of the human body locally, has motivated learning, has promoted students to change from passive memory to thinking, and has improved the learning effect and satisfaction [18–22].

Many knowledge points described in sectional anatomy theory classes need to be further observed in experimental classes. In traditional experimental teaching, students learn through a two-dimensional atlas and slices of specimens. However, the tomographic structure is closely related to the 3D spatial relationships of anatomical structures. In the classroom, it is crucial to find ways to pique the curiosity of learners and help them visualize abstract theoretical concepts. In this study, we made adequate preparations for the control group, but the teaching was not as effective as that for the study group in the same period. It was difficult to stimulate the interest of students in the control group during class. This demonstrates that traditional teaching methods are insufficient.

The 3D printed specimen of the lung segment used in the study group is a detachable combination model with transparent wrapped material, including structures such as a bronchial tree, pulmonary artery, pulmonary vein, and lung profile, with the pulmonary artery in blue and the pulmonary vein in red; each lung segment bronchus is represented by different colored materials, and the lung surface and its filling consist of a transparent, wrapped material. The 3D-printed specimens were colorful and eye-catching. The students showed great interest in them, which created an active class climate. The results showed that the 3D printed specimens of lung segments could effectively aid students in comprehending the ducts within lung segments, as well as the anatomy and CT tomography. The model can facilitate the development of lung segment tomographic thinking skills, enhance teaching satisfaction, and improve the course grade excellence rate. However, there are shortcomings. There are 240 students in third-year majoring in medical imaging in our college, and the high cost of printing materials makes it difficult to provide enough specimens for all students at the same time. We will seek funding support, and we expect the cost of 3D printing materials to decrease in the future to meet the experimental needs. Soft materials cannot be used to print colorful intrapulmonary tubes, so we had to use rigid materials to create models for observation. We expect the specimens of lung segments to be printed with soft materials, which can be sectioned to supplant cadaveric specimens. This will provide a better experimental teaching effect.

## Conclusion

The application of high-precision multicolor 3D printed specimens of lung segments in experimental teaching of sectional anatomy can improve teaching effectiveness and is worth adopting and promoting in sectional anatomy courses.

## Data Availability

The data are available from Shandong Digital Human Technology Co., Inc. To obtain the data from this study, please contact Mr. Xiang Zhou at zx_1313@126.com or Mr. Huachun Miao at mhc@wnmc.edu.cn.

## Abbreviations

3D: Three-dimensional
CT: Computed tomography
MRI: magnetic resonance imaging
VHP: Visual Human Project
CDVH: China’s Digitalized Visible Human
VR: virtual reality.
